# Inferior herbivorous competitors avoid traces of superior competitors to prevent losing competition

**DOI:** 10.1007/s10493-026-01150-x

**Published:** 2026-06-03

**Authors:** Shiori Kinto, Shuichi Yano

**Affiliations:** Kyoto University, Kyoto Prefecture, Kyoto, 606-8502 Japan

**Keywords:** Spider mites, Amensalism, Competition avoidance, Traces avoidance, Competitive exclusion

## Abstract

Species sharing overlapping ecological niches are expected to experience unstable coexistence due to interspecific competition. Non-web-building *Panonychus* spider mites share host plant species with most web-building *Tetranychus* spider mites. Previous research demonstrated that *Panonychus* mites are hindered by the three-dimensional webs produced by *Tetranychus* species, and may experience competitive exclusion. In this study, fecundity of *Panonychus citri* was found to be reduced on leaves bearing *Tetranychus kanzawai* webs, indicating that the webs impose a fitness cost on *P. citri*. Such selective pressure would favor behaviors that allow *Panonychus* mites to avoid direct competition. Consistent with this prediction, migrating *P. citri* avoided *T. kanzawai* traces at a bifurcation, suggesting that they can detect and evade the presence of superior competitors. Moreover, *P. citri* avoided settling onto leaf surfaces with *T. kanzawai* traces including silk threads but no feces or eggs, indicating the ability to recognize competitor establishment from traces on the leaf. This study provides the first evidence that spider mite traces can have a repellent effect on competing heterospecific spider mites. In contrast, fecundity of *T. kanzawai* was not influenced by the presence of *P. citri* webs, and *T. kanzawai* did not avoided *P. citri* traces either at bifurcation or on leaf surfaces. These results suggest that only inferior competitors avoid encountering superior competitors to prevent the amensalistic competitive relationship between them.

**Supplementary Information:**

The online version contains supplementary material available at 10.1007/s10493-026-01150-x.

## Introduction

The coexistence of species tends to be unstable, when they share overlapping ecological niches and often compete for similar resources (Gause 2019). Less competitive species are therefore expected to develop behavioral or ecological mechanisms that minimize direct interference with superior competitors. Such niche adjustment can lead to partial segregation in habitat or food use. Interspecific competition can severely reduce the fitness of inferior competitors and, in extreme cases, result in their competitive exclusion (Reitz and Trumble 2002; Paini et al. 2008). Therefore, the ability to avoid or mitigate interspecific competition in advance should confer a selective advantage to inferior competitors.

The kanzawa spider mite *Tetranychus kanzawai* Kishida, which feed on a variety of wild and cultivated plant species, construct three-dimensional protective webs on host plant leaves and typically resides beneath them (Gerson 1979; Saito 1983). The webs serve as a defensive shelter against generalist predatory mites and ants unable to penetrate them, and as an emergency evacuation site against specialist predators which intrude into the webs (Sabelis and Bakker 1992; Grostal and Dicke 1999; Otsuki and Yano 2014). By contrast, the citrus red mite *Panonychus citri* McGregor, which also utilize a variety of plant species, does not form such complex webs (Saito 1983) but defend itself from predators with defensive setae directed in all upper directions instead of building protective webs (Yano and Shirotsuka 2013). Because the two spider mite species partially share habitats and food plant species such as peach *Prunus persica* L., Batsch, grapevine *Vitis vinifera* L. and lemon *Citrus limon* Burm. (van de Vrie et al. 1972; Bolland et al. 1998), they may come across each other on these plants. Although a previous experiment in orchard has revealed that *Panonychus* spider mites are hindered by three-dimensional webs constructed by *Tetranychus* spider mites and are competitively excluded (Morimoto et al. 2006; Osakabe et al. 2006). Osakabe et al. (2006) demonstrated in field experiments that *Tetranychus* mites’ invasion drove *Panonychus* mites toward upper leaf surfaces, although the mechanisms underlying this distribution shift remains unclear. Therefore, we hypothesized that *P. citri* should have behavioral strategies to avoid encountering nearby *T. kanzawai* and thereby reduce the high costs of interspecific competition.

## Materials and methods

### Spider mites

Both study populations of *P. citri* and *T. kanzawai* were collected from the same trifoliate orange trees *Poncirus trifoliata* L. (Raf.), in Kyoto in 2018. Each species was maintained separately on leaf discs of kidney bean (*Phaseolus vulgaris* L.) pressed on water-saturated cotton in Petri dishes (90 mm diameter, 14 mm depth). These dishes were kept in transparent plastic containers under laboratory conditions (25 ± 2 °C, 50 ± 5% RH, and a 16 h L:8 h D photoperiod). All experiments were conducted under these conditions. Mated adult females were used throughout the study, as they are considered the dispersal stage of spider mites (Gerson 2003; Oku et al. 2005).

### Predatory mites

The generalist predatory mite *Euseius sojaensis* Ehara feeds on both *T. kanzawai* and *P. citri* (Ozawa and Yano 2009; Tsuchida and Masui 2025). The study population of *E. sojaensis* was collected from the kudzu vine *Pueraria lobata* (Willd) Ohwi, in Kyoto in 2009. The population was maintained on tea pollen on leaf discs under laboratory conditions in the same manner as described above. Only mated adult females were used in the experiments.

### Plants

Expanded primary leaves of 10–14-day-old kidney bean plants (*P. vulgaris* cv. ‘Nagauzura’) were used in the following tests. The plants were reared in plastic pots (12 cm diameter, 10 cm depth) under laboratory conditions. This plant species has been reported to be a preferred food for both spider mite species (Ashihara 1987; Oku et al. 2005).

### Effect of spider mite webs on the performance of competitors

**Fig. 1:**
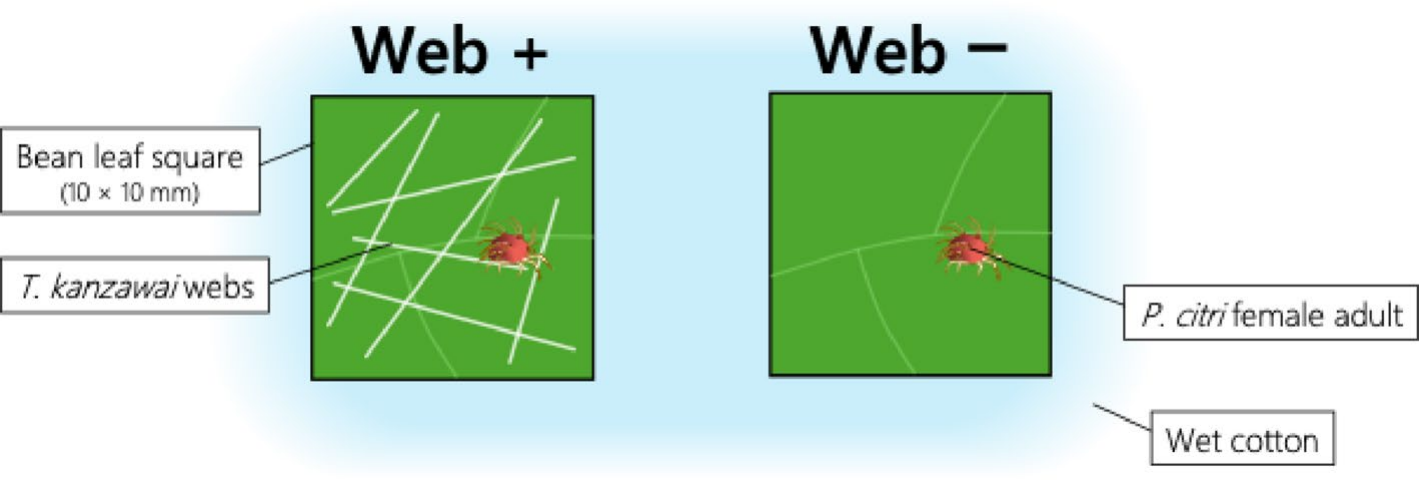
Experimental setup used to observe effect of heterospecific spider mite webs on the number of eggs laid on leaves

Experimental procedures were partly based on those described by Kinto et al. (Kinto et al. 2023), with modifications to test interspecific competition between *P. citri* and *T. kanzawai*. To assess whether webs constructed by *T. kanzawai* on food plant leaves affect the performance of *P. citri*, a 2–4-day-old mated adult female *T. kanzawai* was introduced onto a 10 × 10 mm bean leaf square. Since we had preliminarily confirmed that all 1-day old adult female spider mites had already mated (i.e. produced fertilized eggs) when reared at a density of approximately 50 females and 10 males per 60 mm diameter leaf disc, we assumed that the 2–4-day-old female spider mites had also mated. After 48 h, when *T. kanzawai* had established webs (Yano 2012), all females were carefully removed with minimal damage to the webs. We removed as much of the webs as possible, along with eggs and feces, from half of the leaves (web – condition) using a fine brush. The other half of the leaves with intact webs served as the web + condition. While *T. kanzawai* webs were prepared as described above, newly emerged *P. citri* females were simultaneously prepared as follows. Approximately 40 quiescent female deutonymphs (quiescent stage immediately before adult emergence) of *P. citri* were introduced onto a kidney bean leaf disk (approximately 60 mm in diameter). Immediately after synchronized adult emergence (see Ikegami et al. 2000 for detailed methods), ca. 60 adult males were introduced to allow mating. After 24 h, females were transferred singly onto the prepared leaf squares immediately after the removal of *T. kanzawai* females as described above (Fig. 1). Because the number of eggs laid within a given period is considered the most sensitive performance index of spider mite females (Gotoh et al. 1999; Agrawal 2000), any interference caused by *T. kanzawai* webs should result in fewer eggs laid by *P. citri* females. After 48 h, the eggs laid on the leaf squares were counted. For the web + and web– conditions, 19 and 20 replicates were conducted, respectively. The numbers of *P. citri* eggs laid on leaf squares with and without *T. kanzawai* webs were compared using a generalized linear model (GLM). To evaluate overdispersion, we calculated the dispersion parameter (the ratio of residual deviance to degrees of freedom). Since overdispersion was detected (dispersion parameter > 1.2), a quasi-Poisson distribution was employed for the analysis. These analyses were performed in R version 4.5.2 (R Core Team 2025).

Using the same procedure, the effect of *P. citri* webs on the number of *T. kanzawai* eggs laid on bean leaf squares was examined. Twenty-four replicates were conducted for each of the web + and web– conditions.

### Avoidance of spider mite traces by competitors on migration route

**Fig. 2:**
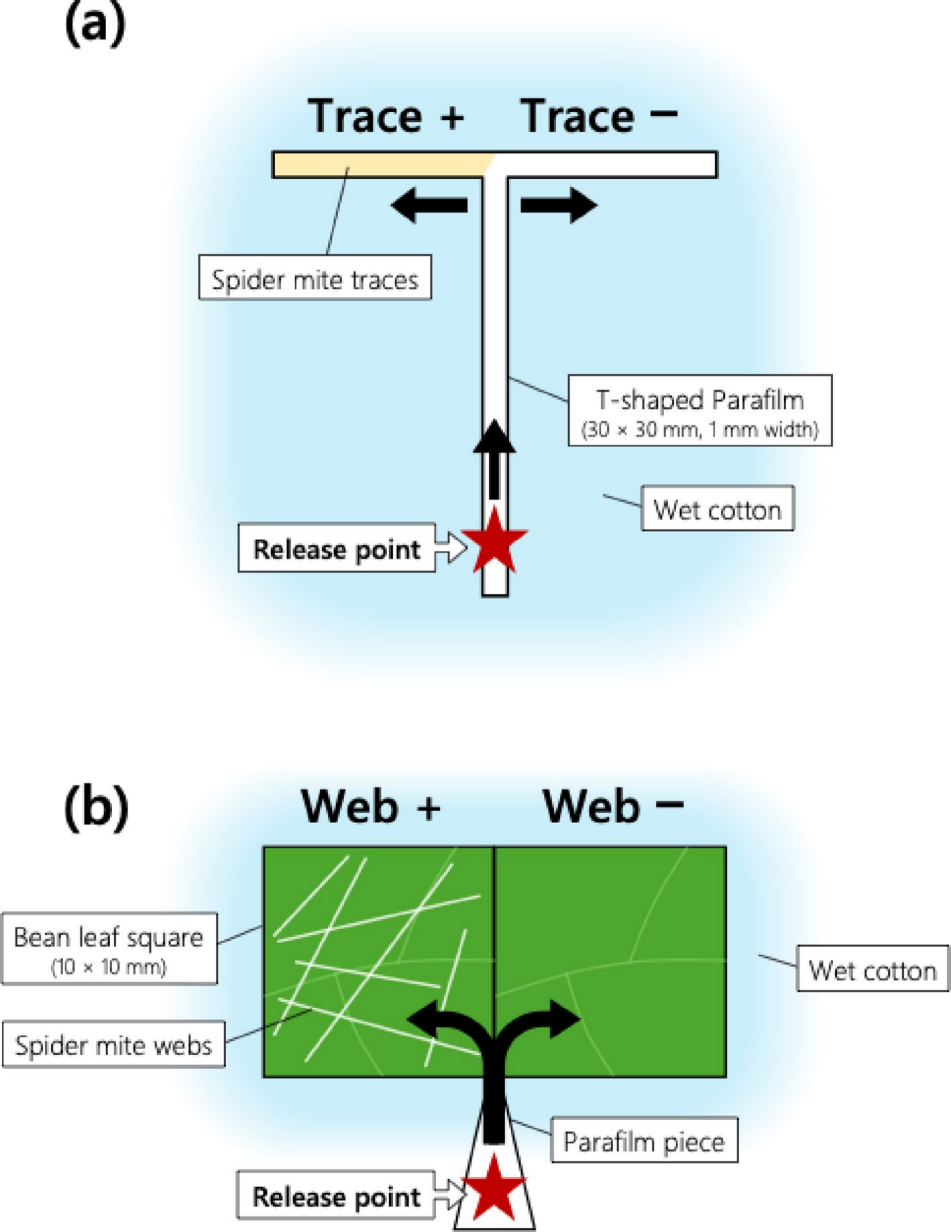
Experimental setup used to observe avoidance of heterospecific spider mite traces on **(a)** T-shaped Parafilm pathway and **(b)** kidney bean leaf surfaces

To determine whether migrating *P. citri* females avoid *T. kanzawai* traces, dual-choice experiments were conducted. The term “trace” as used here refers to silk threads and footprints, but does not include signs of habitation such as feces, eggs, or feeding scars. Two bean leaf squares (10 × 10 mm each) were connected by a T-shaped Parafilm pathway (30 × 30 mm; width 1 mm) on water-saturated cotton in each Petri dish (90 mm diameter, 14 mm depth). To induce *T. kanzawai* traces, one alternately selected branch of the pathway was blocked with a piece of wet filter paper, and then five mated adult female *T. kanzawai* (2–4 days old) were introduced onto a pointed Parafilm piece in contact with the bifurcation to induce traces of five female from the bifurcation to the available leaf square (Fig. 2a). After all females reached leaf squares, the leaf squares, Parafilm pieces, and filter paper were removed. A mated adult female *P. citri* (2–4 days old) was then released at the bottom of the T-shaped pathway (Fig. 2a). The branch chosen by the female to reach the far end was recorded. All females reached one of the far ends within 30 min. Each spider mite and Parafilm setup was used only once. All tests were conducted between 13:00 and 17:00 h, when female adult spider mites actively migrated by walking. Twenty-nine replicates were conducted. Data were analyzed using a two-tailed binomial test using R version 4.5.2 (R Core Team 2025) with the common null hypothesis that mites would choose the both branches with equal probability (i.e., 0.5).

The same procedure was used to examine the response of *T. kanzawai* females to *P. citri* traces on migrating route. Two mandarin orange leaf squares (10 × 10 mm) were connected by a T-shaped Parafilm pathway in the same manner as described above. *P. citri* traces were introduced to one branch of the pathway, and the responses of *T. kanzawai* females to *P. citri* traces were tested in the same manner described above. Thirty-four replicates were conducted.

### Avoidance of spider mite traces by competitors on leaf surfaces

To examine whether *P. citri* females avoid settling on leaves bearing *T. kanzawai* traces, dual-choice experiments were conducted using paired adjacent leaf squares with and without *T. kanzawai* webs. A 10 × 20 mm leaf piece of a primary kidney bean leaf was cut and divided into two equal squares (10 × 10 mm). One square was placed on water-saturated cotton, and a mated adult female *T. kanzawai* (2–4 days old) was introduced onto the square and allowed to construct webs for 30 min. During this period, the female was gently encouraged to keep walking with a fine brush to prevent feeding, oviposition, and excretion on the leaf surface. Since this task exhausts the spider mites, it was practically impossible to continue for more than 30 min. Afterward, the female was carefully removed with minimal damage to the webs. The web-bearing leaf square (web +) was then arranged to touch against the other square (web –) on water-saturated cotton in a Petri dish. A mated adult female *P. citri* (2–4 days old) was subsequently released on a pointed piece of Parafilm in contact with both leaf edges (Fig. 2b). After 2 h, the leaf square on which the mite had settled was recorded, as preliminary observations confirmed that all females would settle on a particular leaf within this period. Each female mite and pair of leaf squares were used only once. Fifty-eight replicates were conducted. Data were subjected to a two-tailed binomial test using R version 4.5.2 (R Core Team 2025) with the common null hypothesis that mites would choose the both leaf squares with equal probability (i.e., 0.5).

The same procedure was used to examine whether *T. kanzawai* females avoided settling on leaves bearing *P. citri* traces. Thirty replicates were conducted. All data analyses were conducted blind with respect to treatment groups.

## Results

### Effect of spider mite webs on the performance of competitors

**Fig. 3:**
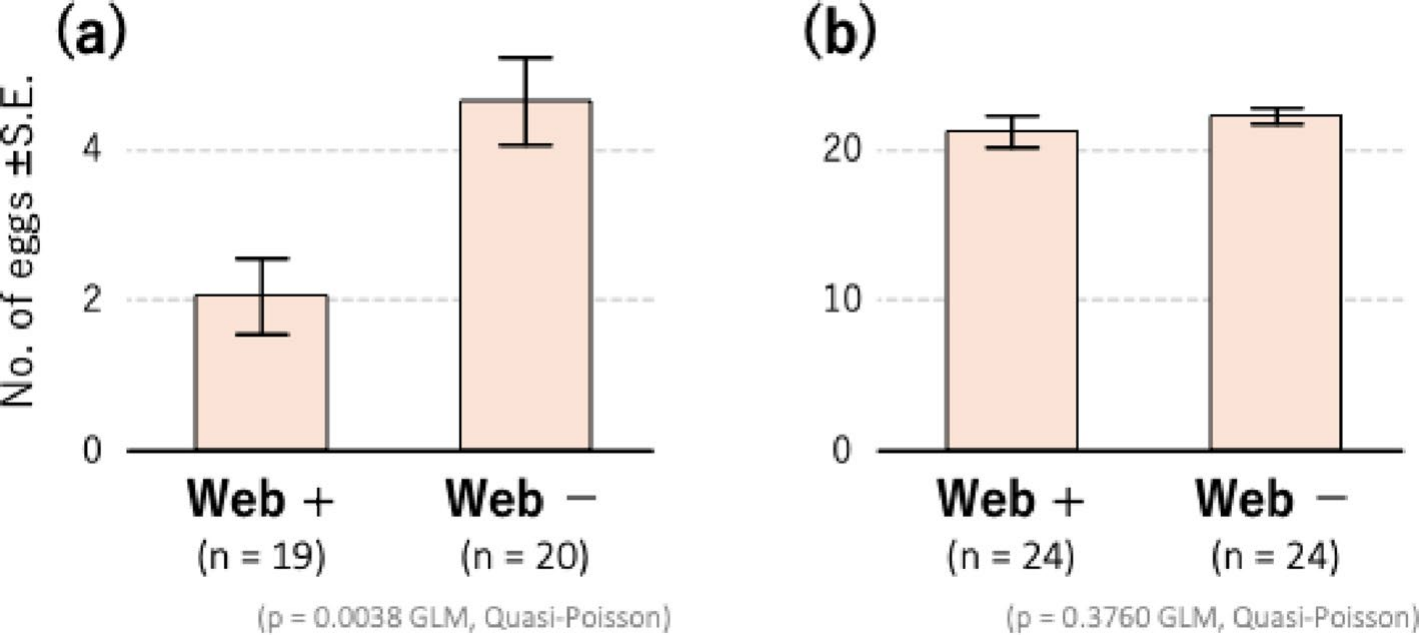
Effects of heterospecific webs on the fecundity of spider mites. Mean (± SE) number of eggs laid per female are shown for (**a**) *Panonychus citri* on leaf squares with or without *Tetranychus kanzawai* webs, and (**b**) *T. kanzawai* on leaf squares with or without *P. citri* webs

The number of eggs laid on the leaves with *T. kanzawai* webs (mean ± standard error [SE] = 2.053 ± 0.5019, *n* = 19) was significantly fewer than on the leaves without webs (4.650 ± 0.5580, *n* = 20; GLM quasi-Poisson, t = − 3.092, *p* = 0.0038; Fig. 3a), suggesting that the presence of *T. kanzawai* webs interferes with the feeding and/or oviposition of *P. citri*.

In contrast, the number of *T. kanzawai* eggs laid on leaf squares with *P. citri* webs (mean ± standard error [SE] = 21.25 ± 1.055, *n* = 24) did not differ significantly with that laid on leaf squares without the webs (22.33 ± 0.5226, *n* = 24; GLM quasi-Poisson, t = − 0.894, *p* = 0.3760; Fig. 3b), suggesting that the presence of *P. citri* webs does not affect the feeding and/or oviposition of *T. kanzawai*. In addition, the number of eggs laid by *T. kanzawai* far exceeded that of *P. citri*, indicating that *T. kanzawai* is more competitive than *P. citri*.

### Avoidance of spider mite traces by competitors on migrating route

**Fig. 4:**
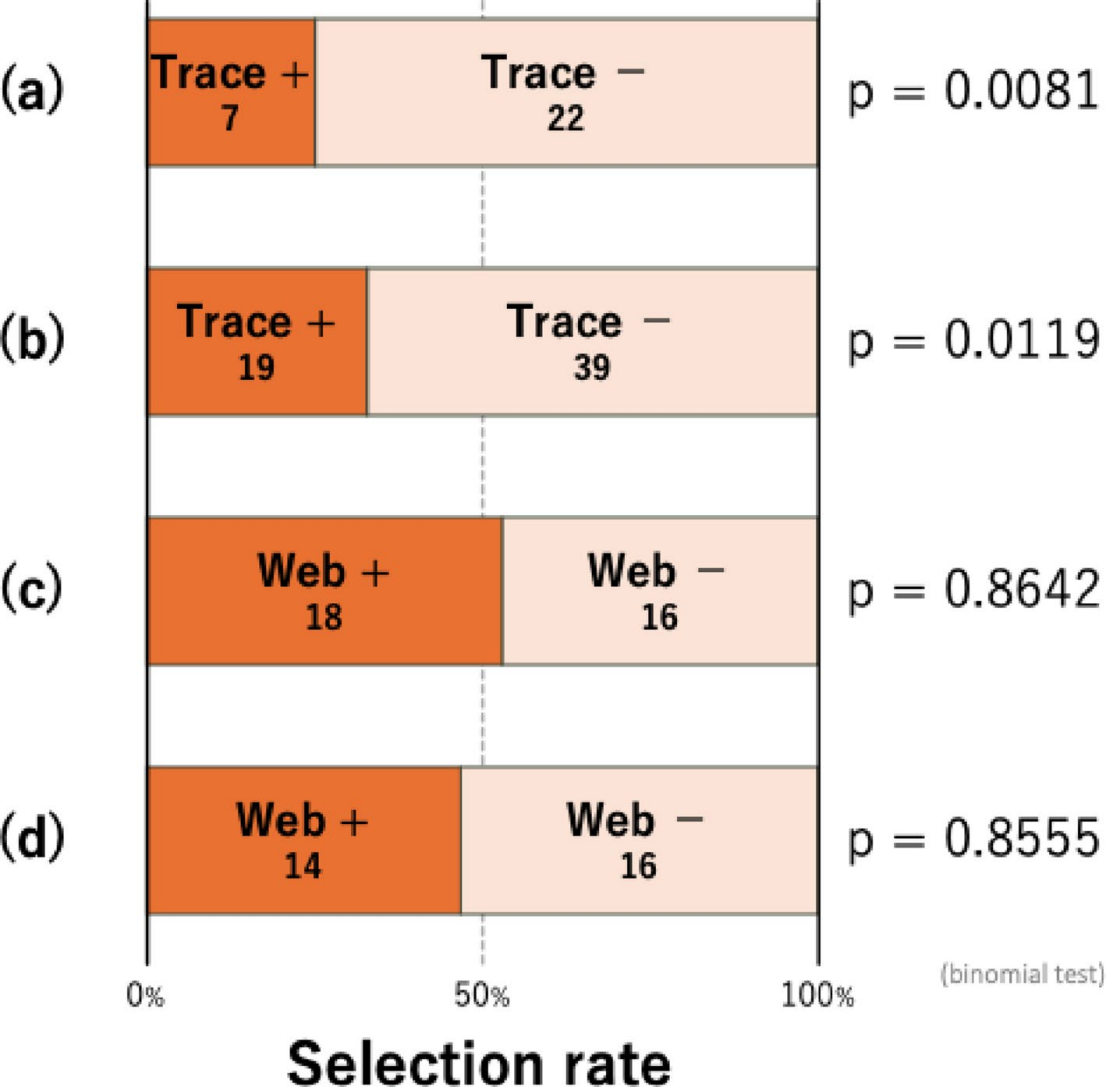
Avoidance of spider mites to heterospecific traces. Avoidance of *P. citri* to *T. kanzawai* traces are shown in (**a**) and (**b**), while avoidance of *T. kanzawai* to *P. citri* traces are shown in (**c**) and (**d**). Panels (**a**) and (**c**) represent the number of females that chose the branch with or without heterospecific traces at a bifurcation on a T-shaped Parafilm pathway. Panels (**b**) and (**d**) represent the number of females that settled on leaf squares with or without heterospecific traces. Numbers within each bar indicate the number of tested females that made each choice

Significantly fewer *P. citri* females walked along branches bearing *T. kanzawai* traces than along branches without traces (binomial test *p* = 0.0081, Fig. 4a), indicating that *P. citri* avoided walking along *T. kanzawai* traces.

In contrast, the number of *T. kanzawai* that walked along the branches did not differ significantly with respect to the presence of *P. citri* traces (binomial test *p* = 0.8642, Fig. 4c), suggesting that *P. citri* traces did not affect migrating *T. kanzawai* females.

### Avoidance of spider mite traces by competitors on leaf surfaces

Significantly fewer *P. citri* females settled onto bean leaf squares with webs of *T. kanzawai* than on squares without webs (binomial test *p* = 0.0119, Fig. 4b), indicating that *P. citri* avoided settling on leaf surfaces with *T. kanzawai* webs.

In contrast, the number of *T. kanzawai* that settled on leaf squares did not differ significantly with respect to the presence of *P. citri* webs (binomial test *p* = 0.8555, Fig. 4d), suggesting that *P. citri* webs did not affect settlement of *T. kanzawai* females on food plant leaves.

## Discussion

*Panonychus citri* laid significantly fewer eggs when placed on leaves bearing *T. kanzawai* webs than on leaves without webs. As other factors such as feeding damage, eggs, and excretions on leaf surfaces were equivalent between the web + and web– treatments, it is likely that *T. kanzawai* webs directly prevented *P. citri* females from laying eggs. This is the first demonstration that spider mite webs reduce the performance of competing species. In contrast, egg numbers of *T. kanzawai* showed no significant difference regardless of whether *P. citri* webs were present. Similar amensalistic relationships between *Panonychus* and *Tetranychus* spider mites have been reported by Osakabe et al. (2006) and Morimoto et al. (2006). The mechanism that causes this relationship may be attributed to web types: webs of *Panonychus* spider mites are classified as little web type (LW; Saito 1983), while webs of *Tetranychus* mites are classified as complicated web type (CW). Although *P. citri* females do not construct three-dimensional (i.e. complicated) protective webs, they protect their eggs by weaving egg guy ropes (Saito 1983). Therefore, complicated *T. kanzawai* webs may have hindered the rope works by *P. citri* females. Furthermore, as shown in the Results section, the fact that *T. kanzawai* lays far more eggs than *P. citri*. could be another reason why *P. citri* should avoid *T. kanzawai*, which is more competitive than itself.

*Panonychus citri* avoided *T. kanzawai* traces at the bifurcation on migrating route, indicating that *P. citri* females prevent the direction chosen by superior competitors during ambulatory migration. Although Pallini et al. (1997) reported that some spider mites avoid the odor of plants infested with heterospecific competitors, this study is the first demonstration that spider mite traces can repel heterospecific spider mite. While previous studies showed that competitors of spider mites avoid webbed leaves (e.g., Pallini et al. 1997), it was unclear whether they avoided the webs or other factors such as feeding-induced host changes. By using artificial Parafilm, we isolated the effect of spider mite traces (silks and footprints) and demonstrated that they have a repellent effect, independent of changes in host plants caused by spider mites. Moreover, *P. citri* also avoided settling on leaves bearing *T. kanzawai* traces, which would in turn prevent the mites from living together with superior competitors on the same leaf. As we carefully prevented *T. kanzawai* females from feeding, ovipositing and excreting during the trace induction, we can reasonably conclude that the avoidance effect seems to be attributed to the webs and/or trace chemicals of *T. kanzawai*.

Previous research showed that some *Tetranychus* spider mites avoid traces of overwhelmingly dominant predators (ants; Yano et al. 2022) and many incidental intraguild predators (caterpillars; Kinto et al. 2023). Although we have yet examined whether *P. citri* avoid such traces along with traces of superior competitors, survival of *P. citri* in the wild seems quite challenging.

Conversely, *T. kanzawai* showed no tendency to avoid *P. citri* traces, suggesting that the trace avoidance is a one-sided response. By avoiding traces of superior competitors, inferior competitors can avoid the one-sided disadvantageous competition at the cost of unrestricted use of food plant resources. The upper leaf surfaces are directly exposed to ultraviolet light and rainfall (Jeppson et al. 1975; Sudo and Osakabe 2011), and are rarely used by the superior competitors, *Tetranychus* spider mites (Foott 1963; Morimoto et al. 2006; Osakabe et al. 2006). Therefore, by examining the costs and benefits of plant use by *Panonychus* mites may provide an answer to why they often prefer upper leaf surfaces (Jones and Parrella 1984; Yano and Shirotsuka 2013). Thus, our results suggest that the selection of seemingly suboptimal habitats and host plants by phytophagous arthropods (Thompson 1988) can be at least partially explained as an adaptive strategy to avoid superior competitors.

## Supplementary Information

Below is the link to the electronic supplementary material.

Supplementary Material 1

## Data Availability

All data produced from this study are provided in this manuscript and supplementary material.
